# Real-Life Experience in the Efficacy and Safety of COVID-19 Vaccination in Patients with Advanced Cirrhosis

**DOI:** 10.3390/jcm12247578

**Published:** 2023-12-08

**Authors:** Amr Shaaban Hanafy, Ahmed Embaby, Sara Mohamed Salem, Ahmed Behiry, Hasnaa Ali Ebrahim, Hany Ahmed Elkattawy, Sally Yussef Abed, Moneer E. Almadani, Mohamad El-Sherbiny

**Affiliations:** 1 Internal Medicine Department, Gastroenterology and Hepatology Division, Zagazig University, Zagazig 44519, Egypt; amrhanafy@zu.edu.eg (A.S.H.); ssalem@medicine.zu.edu.eg (S.M.S.); 2 Clinical Hematology Unit, Internal Medicine Department, Faculty of Medicine, Zagazig University, Zagazig 44519, Egypt; dr.embaby@zu.edu.eg; 3 Department of Tropical Medicine and Endemic Diseases, College of Medicine, Zagazig University, Zagazig 44519, Egypt; asbehery@medicine.zu.edu.eg; 4 Department of Basic Medical Sciences, College of Medicine, Princess Nourah bint Abdulrahman University, P.O. Box 84428, Riyadh 11671, Saudi Arabia; haebrahim@pnu.edu.sa; 5 Department of Basic Medical Sciences, College of Medicine, AlMaarefa University, Riyadh 11597, Saudi Arabia; hmohammed@um.edu.sa; 6 Medical Physiology Department, College of Medicine, Zagazig University, Zagazig 44519, Egypt; 7 Department of Respiratory Care, College of Applied Medical Science in Jubail, Imam Abdulrahman Bin Faisal University, Jubail 34212, Saudi Arabia; syabed@iau.edu.sa; 8 Tropical Medicine Department, Faculty of Medicine, Mansoura University, Mansoura 35516, Egypt; 9 Department of Clinical Medical Sciences, College of Medicine, AlMaarefa University, Riyadh 11597, Saudi Arabia; mmadani@um.edu.sa

**Keywords:** COVID-19, vaccines, complications, MELD, liver cirrhosis, liver volume

## Abstract

COVID-19 infections accelerate liver decompensation and serious liver-related co-morbidities. The aim is to evaluate the safety and impact of COVID vaccines on hepatic disease progression in patients with advanced liver disease and to identify parameters that predict the occurrence of complications. The study involved 70 patients with advanced liver disease who were vaccinated with different COVID vaccines from January 2021 to April 2022. They were evaluated clinically. The laboratory investigation included a complete blood count, liver and kidney function tests, calculation of CTP and MELD scores, plasma levels of ammonia, abdominal ultrasound, and upper GI endoscopy. Twenty patients had experienced complications 64 ± 12 days from the last dose of a vaccination. Twenty patients (28.6%) developed hepatic decompensation and hypothyroidism (*n* = 11, 15.7%), and five (7.14%) patients developed splanchnic thrombosis. There were no COVID-19 reinfections except for two patients who received Sinopharm and developed vaccine-associated enhanced disease (2.9%). Complications after COVID vaccinations were correlated with ALT (r = 0.279, *p* = 0.019), serum sodium (r = −0.30, *p* = 0.005), creatinine (r = 0.303, *p* = 0.011), liver volume (LV) (r = −0.640, *p* = 0.000), and MELD score (r = 0.439, *p* = 0.000). Multivariate logistic regression revealed that LV is the only independent predictor (*p* = 0.001). LV ≤ 682.3 has a sensitivity of 95.24% and a specificity of 85.71% in predicting complications with an AUC of 0.935, *p* < 0.001. In conclusion, the hepatic reserve and prognosis in liver cirrhosis should be evaluated prior to COVID vaccinations using the MELD score and liver volume as promising risk stratification criteria. In summary, the research proposes a novel triaging strategy that involves utilizing the MELD score and liver volume as risk stratification parameters of the hepatic reserve and prognosis of advanced liver cirrhosis prior to COVID immunization to determine who should not receive a COVID vaccination.

## 1. Introduction

The World Health Organization (WHO) declared coronavirus disease-2019 (COVID-19) an international emergency and a worldwide pandemic on 11 March 2020. The condition spreads quickly, making many patients with underlying chronic liver disorders more vulnerable to infection. From a hepatology perspective, COVID-19 management and prevention are currently evolving as it is a relatively new illness [1,2,3].

Pathogenic coronaviruses are enveloped, positive-sense (the sequence is translated directly into proteins), single-stranded RNA viruses that cause respiratory tract infections, ranging from mild to serious, potentially leading to fatal illness [4].

Because angiotensin-converting enzyme 2 (ACE2) represents a functional receptor for coronaviruses through interactions with viral spike proteins, facilitating viral entry, its blockage may interfere with the viral invasion [5]. This viral protein–host receptor interaction represents a potential target for a COVID vaccine-mediated immune response [6].

Nucleoside-modified mRNA-lipid nanoparticle-encapsulated vaccines are one type of vaccination platform in which the SARS CoV-2 spike protein is ingested by host cells, including antigen-presenting cells. The target antigen is produced by the given mRNA using the host ribosomal translational units, which in turn activate cytotoxic CD8 T-cells, helper T-cells, and B-cells to produce a neutralizing antibody response [7,8,9,10]. mRNA vaccines are susceptible to enzymatic catabolism and innate immune-mediated inhibition of mRNA translation, but these challenges have been overcome by using lipid nanoparticles [11]. Polyethylene glycol is used for the stability of the lipid nanoparticles, which may occasionally result in anaphylaxis [12].

Pfizer/BioNTech BNT162b2 mRNA vaccine and Moderna are two examples of mRNA vaccines (mRNA-12730). The Pfizer-BioNTech vaccine is administered in two doses of 30 μg, 0.3 mL, three weeks apart. Seven days following the second dosage, the vaccine’s effectiveness (VE) in preventing infection was 95% [13].

The SARS-CoV-2 spike protein is encoded by DNA that is carried by an exogenous virus that has been genetically modified for use in adenoviral vector vaccines. Patients with impaired immune systems should not be in danger from the adenovirus vector, as it has been altered to stop replication in host cells by removing E1–E3 sections from the viral genome. The adenovirus causes the transcription of the appropriate mRNA once it has entered the cell and delivered the DNA for the SARS-CoV-2 spike protein into the nucleus [14].

The adenovirus ChAdOx1 nCoV-19 vaccine from Oxford/AstraZeneca is one example of an adenoviral vaccine; fourteen days after the second dosage, the VE is 70.4% [15]. The Janssen vaccine (Johnson & Johnson, Ad26.COV2), which is based on an adenovirus 26 that is replication-incompetent and expresses a stabilized spike protein, has VE rates of 57 to 72% fourteen days after 10 × 5 virons (0.5 mL) are administered [16]. Adenovirus 26 is used as the first replication-incompetent vector dosage in the Sputnik vaccine, and 21 days to 3 months later, an adenovirus 5 vector boosting dose is used [17]. The non-replicating adenovirus-5-vectored inhalation vaccination from CanSino is being used in emergencies in China [18].

SARS-CoV-2 was used to create inactivated whole-viral vaccines, which were then chemically inactivated using formalin or heat after being cultured in cell cultures. The Sinopharm vaccine (WIV04 and HB02), an inactivated whole virus vaccine derived from two distinct strains, is one of its components. Given in two intramuscular (IM) doses of 0.5 mL each, separated by 2–4 weeks, it had a VE of 73% for WIV04 and 78% for HB02 fourteen days after the second dose of the vaccination [19]. An unusual adverse effect of inactivated vaccinations known as “vaccine-associated enhanced disease” is defined by eosinophilic lung infiltrations upon viral re-exposure [20].

In COVID-19, the prevalence of chronic liver disease was 3%, according to eleven observational studies including 2034 individuals [21]. In patients receiving critical care, up to 53% had elevated serum transaminases and total bilirubin levels [22,23]. The direct viral cytopathic impact, systemic inflammatory response, immunological hepatopathy, and pre-existing liver disease are the causes of liver injury related to COVID-19 that have been proposed [24]. It was discovered that hepatocytes, sinusoidal endothelium, and cholangiocytes all had high ACE2 levels, which may account for the virus’s affinity to the liver [25].

While prompt COVID vaccination is essential for patients with chronic liver disease to avoid hepatic decompensation in the event of an infection, some patients may experience serious side effects after vaccination. Therefore, the current study aims to confirm the safety of COVID vaccines in patients with advanced liver disease and provide novel risk stratification parameters of the hepatic reserve and prognosis prior to COVID immunization in order to identify patients who should not receive a COVID vaccination.

## 2. Material and Methods

### 2.1. Study Design

#### 2.1.1. Ethical Approval

The study was approved by the Ethics Committee for Human Research guidelines at the College of Medicine, Zagazig University (Approval number: ZU-IRB-#10926-9/7-23).

#### 2.1.2. Patient Selection

This prospective study was conducted from January 2021 to April 2022 at the Department of Internal Medicine, Hepatology Outpatient Clinic, Zagazig University, Egypt. Written informed consent was obtained from each patient prior to their inclusion in the study. The study involved 70 patients with advanced liver disease who were vaccinated through immunization centers and Ministry of Health-led immunization campaigns; the patients were examined 6 months following vaccination for effects on synthetic liver functions and potential adverse effects.

The etiology of chronic liver disease was Hepatitis C virus (HCV) (*n* = 65), and all the patients had received antiviral therapy with sustained virological response and HBV (*n* = 5) with a documented absence of active Hepatitis B virus (HBV) viremia.

Patients were included if they had chronic parenchymatous liver disease with compensated cirrhosis confirmed by abdominal ultrasound and a Child–Pugh score class A or B. The etiology, either HCV or HBV, was diagnosed via HCV-RNA and HBV-DNA using polymerase chain reaction (PCR).

Patients were excluded if they had current parenchymatous hepatic decompensation (ascites, hepatic encephalopathy, and acute liver failure), hepatocellular carcinoma or other extrahepatic malignancy, or known hypersensitivity to the vaccines, and patients who refused to participate in the study.

#### 2.1.3. Patients’ Evaluation

Every patient had a thorough history taken, a clinical examination for signs of decompensated liver cirrhosis, and other systems examinations.

#### 2.1.4. Laboratory Investigations 

These included complete blood count (CBC), liver and kidney function tests, prothrombin time and concentration, and international normalized ratio (INR), in addition to the calculation of the CTP and MELD scores. 

Real-time quantitative PCR for HCV-RNA or HBV-DNA was performed for suspected cases with active viremia.

#### 2.1.5. Plasma Level of Ammonia 

A venous blood sample was collected after 6 h of fasting. The samples were put in EDTA-containing sterile tubes, and analysis was performed utilizing the enzymatic method (*n* = 10–60 μg/dL).

#### 2.1.6. Abdominal Ultrasound Evaluation

This was performed by a single, well-experienced physician using an ultrasound device (Sonoscape A6T, China, Shanghai: Heyi Medical Instrument Co., Ltd.). Portal hypertension parameters such as portal vein diameter, splenic bipolar diameter, and splenic vein diameter were evaluated. Patients with ascites, new appearances, or recurrences of hepatocellular carcinoma or vascular thrombosis after a vaccination were recorded.

Adults typically have a liver volume of around 1260 to 1600 mL, according to bedside ultrasonography estimates using the conventional ellipsoid formula (max. length × max. width × max. depth × 0.523) [26].

#### 2.1.7. Upper GI Endoscopy

This procedure was carried out as part of the routine follow-up for high-risk patients with established gastro-esophageal varices, and the status of the varices was recorded following COVID immunization.

#### 2.1.8. Statistical Plan

The results were analyzed using the SPSS version 20 (IBM SPSS Statistics. Chicago, IL, USA: SPSS Inc.). The Shapiro–Wilk test was used to determine if the data were normally distributed. If the *p* value of the test is higher than 0.05, the data are considered normal. The data significantly deviate from a normal distribution if it is less than 0.05. The descriptions of continuous variables were the median, IQR, or mean ± standard deviation. If the data were normally distributed, the independent group t-test was performed to compare the continuous variables; otherwise, the Mann–Whitney U-test was employed. For comparison of paired results before and after vaccination, a paired t-test was carried out, however, the Wilcoxon signed-ranks test was performed as a non-parametric alternative to paired t-test. It is most commonly used to test for a difference in the mean (or median) of paired observations. A Spearman’s correlation coefficient (r) was performed for ordinal variables, with a Pearson correlation for continuous variables. Logistic regression analysis was performed by forward selection to identify variables independently associated with complications and outcomes. All variables with *p* < 0.05 were considered statistically significant.

A receiver operating curve (ROC) was performed to test the diagnostic efficacy of the high-risk variables. The performance of the cutoff was assessed via the calculation of Youden’s J value; Values close to 1 indicated good performance (J = sensitivity + specificity 1)

## 3. Results

Seventy patients with chronic liver disease were recruited for the study, and their follow-up period was extended to six months following the final dosage of the COVID-19 vaccination. The causes of chronic liver disease were HCV (92.9%) and HBV (7.1%) and none of the patients had active viremia.

Forty-four males and twenty-six females were enrolled, with a mean age of 55 ± 12 years; twenty-two patients (31.4%) had diabetes. At baseline, a mild elevation of the mean serum total bilirubin and a mild decrease in the mean serum albumin were observed. Due to liver cirrhosis, there was a mild thrombocytopenia (109 ± 21 × 109/L) with a mean MELD score of 17 ± 3, and an abdominal ultrasound revealed a mean liver volume of 743.9 ± 171.3 mL. Most patients were classified as CTP score class A (forty-five patients, 64.3%, score 5–6) or class B (twenty-five patients, 35.7%, score 7–8). The mean value of FIB-4 was 4.16 ± 1.45, and at baseline, no one showed signs of decompensation, such as ascites or jaundice (Table 1).

Gastro-esophageal varices were found in twenty-eight individuals (40%) during routine endoscopic monitoring for esophageal varices [Grade 1–2 varices, *n* = 18; grade 3 varices, *n* = 5; gastric varices, *n* = 5].

**Figure 1:**
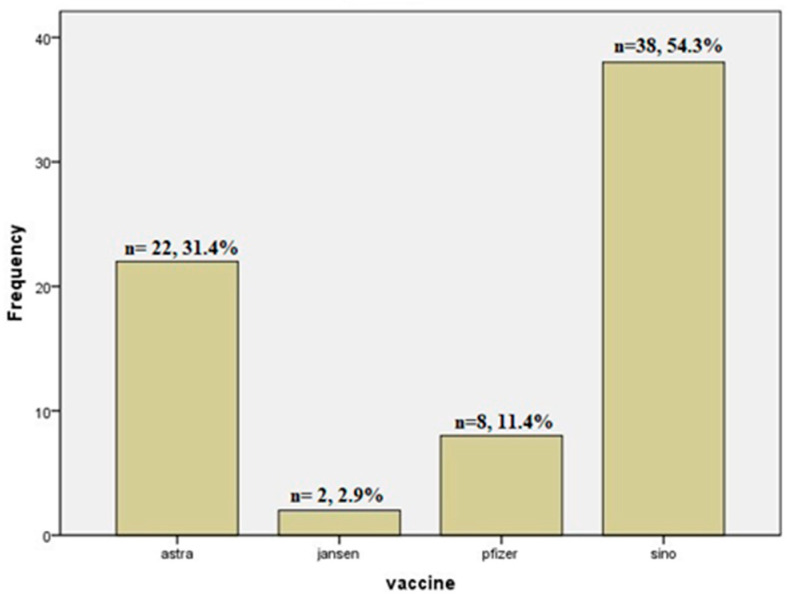
Bar chart showing the different types of administered COVID vaccines among the studied patients.

Mass immunization was encouraged and promoted during the COVID-19 pandemic. Figure 1, Table 1, shows that thirty-eight patients (54.3%) received the inactivated whole virus Sinopharm vaccine in two doses; twenty-two patients (31.4%) received the AstraZeneca adenovirus ChAdOx1 nCoV-19 vaccine in two doses; eight patients (11.4%) received the mRNA Pfizer vaccine in two doses; and two patients (2.9%) received one dose of the Janssen vaccine (Ad26.COV2).

The mean duration from the last vaccine dose to the development of complications was 64 ± 12 days; twenty patients (28.6%) had suffered complications at varying times within the post-vaccination period. Decompensation manifested as mild-to-moderate ascites in 20/20 patients (100%) and led to an increase in total bilirubin and a decrease in serum albumin. Eleven patients (55%) showed signs of hypothyroidism, including weight gain, sluggish thinking, constipation, and elevated TSH (16.9 ± 4.45 mIU/L). Five (25%) patients experienced splanchnic thrombosis; three of them experienced portal thrombosis, and two of them developed a hepatic venous thrombus that spread to the inferior vena cava and resulted in ascites. These patients were treated with enoxaparin and were kept on Rivaroxaban for 4 months.

**Figure 2:**
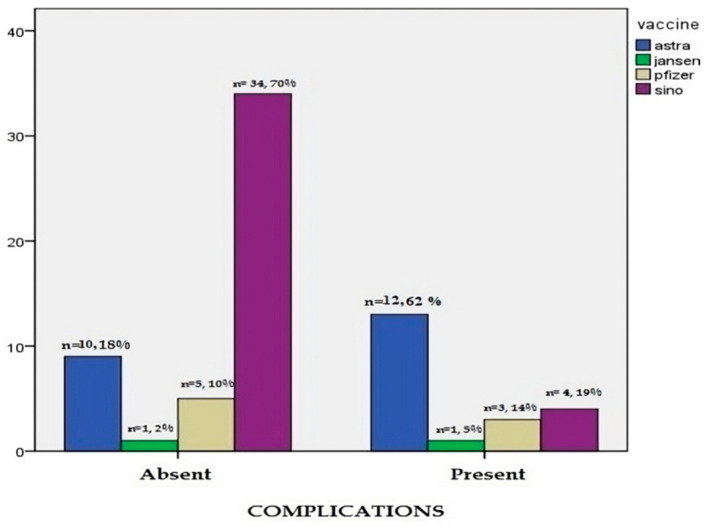
The incidence of the COVID-19 vaccine-related complications.

Cardiomyopathy was evident in 5/20 patients (25%), characterized by dyspnea on exertion and irregular or dropped beats, and echocardiography revealed dilated cardiomyopathy (three patients after Pfizer and two patients after AstraZeneca). Vaccine-associated enhanced disease was shown in two patients (10%) characterized by diffuse lung infiltration with hypoxia after re-exposure to a new COVID-19 infection. The patients gave a previous history of vaccination with the Sinopharm vaccine, and nasal swabs were negative (Table 2, Figure 2).

In the vaccinated cirrhotic patients who experienced post-vaccination complications, a substantial rise in the serum levels of the total bilirubin (*p* = 0.000), INR (*p* = 0.037), and serum ammonia (*p* = 0.02) was observed, with a significant decrease in blood levels of hemoglobin (*p* = 0.038) and albumin (*p* = 0.000) were observed. Additionally, there was a noteworthy rise in the MELD-Na value (*p* = 0.001) with a significant worsening of the CTP class (*p* = 0.000) (Table 3).

Regarding the effect of vaccination on the progression of oesophageal varices, routine endoscopic surveillance in vaccinated individuals did not reveal any changes in the variceal size or the emergence of new varices.

The likelihood of complications following a COVID vaccination in patients with liver cirrhosis was correlated with the following variables: liver volume (r = −0.640, *p* = 0.000), creatinine level (r = 0.302, *p* = 0.011), ALT level (r = 0.279, *p* = 0.019), serum sodium (r = −0.330, *p* = 0.005), and MELD score (r = 0.439, *p* = 0.000). Negative correlations were found with serum sodium and liver volume, as shown in Table 4.

**Figure 3:**
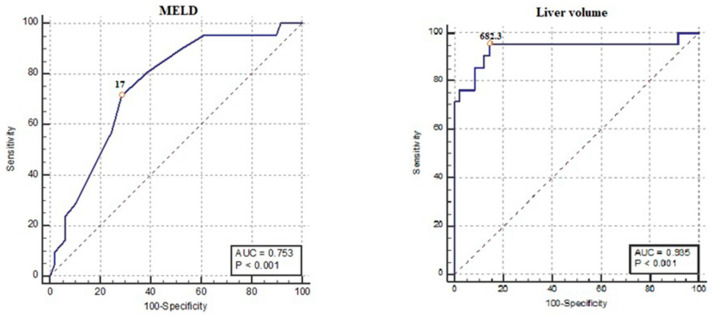
ROC curves demonstrate the liver volume and MELD score cutoff values as predictors of problems following COVID vaccination.

**Figure 4:**
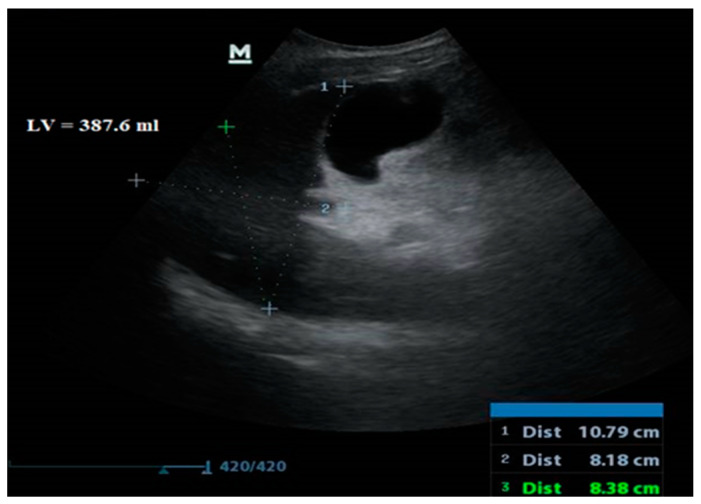
Liver volume measured via abdominal ultrasound.

The receiver operating characteristic curve analysis showed the variables most correlated with complications after COVID vaccinations in cirrhotic patients (*p* = 0.000) were the MELD Score and liver volume, and it revealed that the MELD score at a cutoff value > 17 was associated with complications with a sensitivity and a specificity of 71.4% and 70%, respectively, and an area under curve (AUC) of 0.753, *p* value < 0.001. A liver volume ≤ 682.3 mL has a sensitivity of 95.24% and specificity of 85.71% in predicting complications, with an AUC of 0.935, *p* < 0.001 (Table 5, Figure 3 and Figure 4).

Liver volume is the only independent predictor of post-COVID-19 vaccination complications, according to multivariate logistic regression analysis (*p* = 0.001), with a 95% confidence interval of 0.97–0.99 (Table 6).

## 4. Discussion

The current study’s objective was to examine the practical safety of the COVID vaccinations in a specific subset of chronic liver disease patients with advanced cirrhosis. It also postulated parameters that can identify high-risk individuals who may experience vaccine-related complications. Despite the fact that the vaccination is unquestionably protective against infection-induced progression of chronic liver disease because of immune dysfunction associated with cirrhosis, which speeds up hepatic decompensation and causes variceal hemorrhage and hepatic encephalopathy, some patients may experience increased significant morbidity [27].

Prior HAV, HBV, influenza, and pneumococcal vaccines have been demonstrated to lower hospitalization and death from chronic liver disease, therefore COVID-19 immunization is crucial even when vaccine hyporeactivity is predicted [28].

Given that hospitalized COVID-19 patients with cirrhosis had an all-cause death rate of 38% (about 70% in Child–Pugh C) compared to just 8% in patients without cirrhosis, it is crucial to balance the possible advantages of immunization against the hazards [29,30].

In the current study, the real-life efficacy and safety of COVID-19 vaccines in patients with advanced liver disease were evaluated. No one developed an active COVID-19 re-infection during the period of follow up. However, two patients who were exposed to a COVID-19 infection after the Sinopharm vaccination developed vaccine-associated enhanced disease characterized by intense pulmonary infiltrations and dyspnea with hypoxia, despite the negativity of viral RNA in nasal swabs. This rare phenomenon was reported previously after dengue virus, respiratory syncytial virus, and measles vaccines and could be explained by neutralizing antibody-dependent immune complex deposition in the lungs (Fc-mediated immune cell acquisition, complement receptors-mediated, and Th2-mediated pulmonary eosinophilic hypersensitivity reactions) [31].

Twenty patients (28.6%) experienced hepatic decompensation in the form of ascites and hyperbilirubinemia 63.5 ± 12.1 days after the last vaccination dose. This finding is supported by two earlier reports: one from a study that found 1.2% of COVID vaccine recipients experienced acute decompensation that required hospitalization [32], and another reporting that two cases experienced severe jaundice and pruritus that persisted for a long time after receiving the Pfizer BioNTech COVID-19 mRNA vaccine [33].

This was also seen in a previous case report that examined the histological characteristics of vaccine-induced hepatic damage, including intrahepatic cholestasis and a substantial neutrophil infiltration with Kupffer cells and macrophages loaded with ceroid. Additionally, immunological indicators showed an improvement without the requirement for immunosuppressive drugs [34].

The possible mechanisms of hepatic decompensation are difficult to explain, but aggressive cytokine activation may be the cause, as nearly 40% of patients with chronic liver disease decompensate after cytokine production induced by COVID-19 infection or vaccination, resulting in increased hepatocyte apoptosis and necrosis [35].

Hepatic decompensation was shown to be linked in the current study with baseline levels of ALT (*p* = 0.019), serum sodium (*p* = 0.005), serum creatinine (*p* = 0.01), liver volume (*p* = 0.000), and MELD (*p* = 0.000).

A total of 15.7% of the vaccinated chronic liver disease patients developed thyroid hypofunction 63.6 ± 22.5 days from the dose of vaccination, mainly after the AstraZeneca vaccine (54.4%) and the inactivated Sinopharm vaccine (36.4%). No one had a prior or family history of thyroid illness, and this was significantly correlated with reduced liver volume. This was consistent with previous reports of autoimmune hypothyroidism after a COVID vaccination [36] and another report of sub-acute thyroiditis occurring after vaccination with mRNA-based vaccines (68.7%), viral vector vaccines (15.7%), and 14.5% following inactivated vaccines [37]. A recent study enrolled 2765 patients and revealed that a COVID-19 vaccination seemed to induce increased levels of TSH followed by mood disturbance, and pre-vaccine serum TSH, CRP, and anti-TPO should be evaluated [38]. The molecular similarity between the vaccine antigen and thyroid proteins may cause an autoimmune reaction in genetically predisposed individuals [39].

In the current study, splanchnic thrombosis (ST) occurred in 7.14% of cirrhotic vaccinated patients and in 25% of vaccination-associated complications 71 ± 6.67 days post-vaccination (60% were AstraZeneca vaccines and 40% were Pfizer vaccines), and that was significantly correlated with baseline serum creatinine and liver volume. The previously reported incidence of splanchnic thrombosis in COVID-19 patients was 0.6%, but the data were insufficient to estimate its incidence after a COVID-19 vaccination [40]. Another study confirmed our results and concluded that most of these cases were reported exclusively after vector-based vaccines (AstraZeneca and Janssen vaccines) and less with mRNA vaccines (Pfizer-BioNTech) [41]. Cirrhotic patients have a higher risk of bleeding, but occasionally they may develop paradoxical thrombosis, which is correlated with a low pre-vaccination level of albumin [42], protein C, S, and factor V Leiden mutation [43].

In the current study, cardiomyopathy following COVID vaccination was evident in 7.14% of cirrhotic vaccinated patients and was significantly correlated with liver volume; another study found that the risk of myocardial affection increases following sequential doses, including a booster dose of BNT162b2 mRNA vaccine, and mainly in young obese men [44], and was higher after COVID-19 infection than after vaccination. Liver cirrhosis is linked to an increased risk of cirrhotic cardiomyopathy in 50% of patients, which is characterized by impaired contractility, diastolic dysfunction, a hyperdynamic circulatory state, and QT prolongation [45]. Prior to vaccination in patients with advanced cirrhosis, it is advised to do ECG, calculating the corrected QT interval and echocardiography.

The current study showed that complications after a COVID vaccination were significantly correlated with higher levels of ALT, creatinine, MELD score, a lower sodium level, and reduced liver volume. A liver volume lower than 682.3 mL has a sensitivity of 95.24% and a specificity of 85.71% in predicting complications post-COVID vaccination.

The main strengths of this study are providing for the first time the real-life experience of the efficacy and safety of COVID-19 vaccinations in patients with advanced cirrhosis and shed light on complications that may occur, stratifying predictors of complications to determine patients that should not receive COVID vaccination This is in contrast to a previous Pfizer-BioNTech study that recruited 214 individuals with liver disease out of 37.706 participants (0.6%), three of whom had moderate-to-severe liver disease (0.1%), which is regarded as a small number compared to our study, where the safety and vaccination effectiveness results have not yet been released [46]. Also, in the Janssen phase 3 study, 206 participants (0.5%) with liver disease were included; 103 of them received the vaccination, and interpretation was restricted [47].

### 4.1. Limitations

The primary limitations are that the trial was conducted in a single center and did not assess the vaccinations in other patient groupings that require more investigation, such as those with autoimmune hepatitis or liver transplant recipients.

### 4.2. Future Directions

These findings contribute to our understanding of how COVID vaccination, despite its importance, may alter the clinical course of some patients with advanced liver disease. Triaging patients who may receive the benefit of vaccination and who should not receive vaccination can avoid significantly morbid complications such as hepatic decompensation, hypothyroidism, and splanchnic Thrombosis.

## 5. Conclusions

Although vaccination is crucial for patients with chronic liver disease, this study offered new indicators of hepatic reserve and prognosis in advanced liver disease. These indicators should be assessed before vaccination using the MELD score and liver volume as viable risk stratification criteria in order to prevent serious complications.

## Figures and Tables

**Table 1:** Comparison of demographic and laboratory characteristics based on the presence of COVID-19 vaccine complications.

Parameter		COVID-19 Vaccine Complications		Total		*p*
		Absent	Present		SW, *p*	
		N = 50	N = 20	N = 70		
**Age (years)**		54 ± 11	57 ± 12	**55 (29–87) IQR 15**	0.583	0.335
**Sex**	0	19 (38.8%)	7 (33.3%)	26 (37.1%)		0.666
	1	30 (61.2%)	14 (66.7%)	44 (62.9%)		
**DM**	0	33 (67.3%)	15 (71.4%)	48 (68.6%)		0.736
	1	16 (32.7%)	6 (28.6%)	22 (31.4%)		
**WBC (×10^9^/L)**		5.49 ± 2.35	5.29 ± 1.67	**4.83 (0.93–13.2) IQR 2.67**	**0.003**	0.953
**HB (gm/dL)**		12.2 ± 1.6	12.3 ± 1.1	**12 (10–17) IQR 2.23**	**0.003**	0.398
**PLT (×10^9^/L)**		109 ± 22	110 ± 19	109 ± 21	0.09	0.851
**ALT (IU/L)**		45 ± 11	39 ± 10	43 ± 11	0.282	**0.045**
**AST (IU/L)**		51 ± 10	55 ± 13	52 ± 11	0.361	0.256
**PC%**		56 ± 10	57 ± 11	56 ± 10	0.54	0.645
**INR**		1.58 ± 0.24	1.66 ± 0.27	1.61 ± 0.25	0.414	0.226
**Total Bilirubin (mg/dL)**		1.42 ± 0.31	1.54 ± 0.31	**1.4 (0.88–2.2) IQR 0.45**	**0.041**	0.083
**Albumin (gm/dL)**		3.39 ± 0.48	3.32 ± 0.40	3.37 ± 0.45	0.885	0.576
**Ammonia (ug/dL)**		74.1 ± 17.4	79.1 ± 19.2	75.6 ± 18.0	0.409	0.287
**Sodium (mEq/L)**		140 ± 4	137 ± 5	**139 (130–147) IQR 7.3**	**0.038**	**0.006**
**Creatinine (mg/dL)**		1.32 ± 0.25	1.49 ± 0.25	1.37 ± 0.26	0.937	**0.041**
**Liver Volume (mL)**		808.3 ± 147.8	593.5 ± 122.0	**726 (403–1280) IQR 211**	**0.01**	**<0.001**
**MELD**		16 ± 3	19 ± 3	17 ± 3	0.265	**0.001**
**CTP**	5	17 (34.7%)	4 (19.0%)	21 (30.0%)		0.309
	6	17 (34.7%)	7 (33.3%)	24 (34.3%)		
	7	13 (26.5%)	7 (33.3%)	20 (28.6%)		
	8	2 (4.1%)	3 (14.3%)	5 (7.1%)		
**FIB-4**		3.96 ± 1.31	4.61 ± 1.69	**3.8 (1.4–7.7) IQR 1.64**	**0.000**	0.123
**Vaccine Type**	Astra^®^	10 (18.4%)	12 (61.9%)	22 (31.4%)		**0.001**
	Jansen^®^	1 (2.0%)	1 (4.8%)	2 (2.9%)		
	Pfizer^®^	5 (10.2%)	3 (14.3%)	8 (11.4%)		
	Sino^®^	34 (69.4%)	4 (19.0%)	38 (54.3%)		

Normally distributed quantitative variables were expressed as Mean ± SD and compared using Independent T-test, while non-normally distributed data was expressed as median, IQR, and compared using the Mann–Whitney test. Qualitative variables were expressed as numbers and percentages and compared using the Chi-square X^2^ test. SW, *p*: the *p* value of Shapiro–Wilk test, *p* > 0.05 indicates normally distributed data. Atrazeneca: Oxford University and British-Swedish company AstraZeneca, Cambridge, UK; Jansen: Janssen Vaccines in Leiden, Netherlands; Pfizer: German biotechnology company BioNTech, Mainz, Germany; Sinopharm: China National Pharmaceutical Group Corporation, Beijing, China.

**Table 2:** Incidence, types of COVID-19 vaccine complications, and their corresponding correlated variables.

Complications (*n =* 20)	Age	Sex	Diabetes	ALT	Sodium	Creatinine	MELD	Liver Volume (LV)	Correlation (*p*)
**Decompensation (*n =* 20, 100%)**	56.1 ± 10.3	14M/6F	6 (30%)	38.7 ± 9.3	136.5 ± 4.61	1.5 ± 0.25	19.1 ± 2.5	571.7 ± 71.6	-ALT (0.019)
									-Sodium (0.005)
									-Creatinine (0.01)
									-LV (0.000)
									-MELD (0.000)
**Hypothyroidism (*n =* 11, 55%)**	59.4 ± 6.9	5M/6F	1 (9.1%)	43.1 ± 8.8	139 ± 4.58	1.44 ± 0.37	17.5 ± 3.5	614.3 ± 192	-LV (0.005)
**Splanchnic Thrombosis (*n =* 5, 25%)**	56.5 ± 8.7	3M/2F	3 (60%)	38 ± 9.2	136.2 ± 4.4	1.7 ± 0.33	19.4 ± 3.3	539.1 ± 89.5	-Creatinine (0.003)
									-LV (0.005)
**Cardiomyopathy (*n =* 5, 25%)**	58 ± 8.75	3M/2F	0 (0%)	38.4 ± 8.7	137.4 ± 5.9	1.53 ± 0.3	18.8 ± 1.8	509.7 ± 42.2	-LV (0.001)
**Vaccine-associated enhanced disease (*n =* 2, 10%)**	59.5 ± 10.6	2F	0 (0%)	30 ± 5.7	137 ± 5.65	1.33 ± 0.01	18.5 ± 0.71	531.5 ± 114.8	None
***p***	0.884	-	**0.029**	0.392	0.693	0.5	0.61	0.49	-

**Table 3:** Comparison of pre- and post-vaccination laboratory features of the complication subgroup.

Variable	COVID-19 Vaccine Complications		*p*
	Before Vaccine	After Vaccine	
	N *=* 20	N *=* 20	
**WBC (×10^9^/L)**	5.32 ± 1.7	4.81 ± 0.76	0.324
**Hemoglobin (gm/L)**	12.36 ± 1.07	11.6 ± 1.2	0.038
**Platelets (×10^9^/L)**	108.9 ± 19.2	109.1 ± 8.67	0.97
**INR**	1.67 ± 0.28	1.92 ± 0.42	0.037
**Total Bilirubin (mg/dL)**	1.56 ± 0.31	2.73 ± 0.5	0.000
**Albumin (gm/dL)**	3.33 ± 0.41	2.66 ± 0.29	0.000
**Ammonia (ug/dL)**	79.2 ± 19.7	96 ± 18.8	0.02
**Sodium (mEq/L)**	136.45 ± 4.6	135.6 ± 4.9	0.613
**Creatinine (mg/dL)**	1.49 ± 0.25	1.57 ± 0.36	0.413
**CTP**	6.45 ± 1	9.65 ± 0.75	0.000 **
**MELD**	19.1 ± 2.5	22.9 ± 3.91	0.001

For paired results before and after vaccination, a paired *t*-test for was carried out, however, a Wilcoxon signed-ranks test ** is used as a non-parametric alternative.

**Table 4:** Correlations between examined parameters and complications among vaccinated cirrhotic patients.

	COMPLICATIONS	
	Pearson Correlation	Sig. (2-Tailed)
**Age**	0.096	0.427
**Sex**	0.093	0.441
**DM**	−0.019	0.873
**WBC**	−0.030	0.805
**HB**	−0.046	0.704
**PLT**	−0.008	0.947
**ALT**	0.279	**0.019**
**AST**	0.105	0.385
**PC%**	0.032	0.795
**INR**	0.153	0.205
**Total Bilirubin**	0.204	0.091
**Albumin**	−0.058	0.634
**Ammonia**	0.129	0.288
**Sodium**	−0.330	**0.005**
**Creatinine**	0.302	**0.011**
**Liver Volume**	−0.640	**0.000**
**MELD**	0.439	**0.000**
**CTP**	0.220	0.067
**FIB-4**	0.227	0.058

**Table 5:** The cutoff values of the MELD score and liver volume in predicting COVID-19 vaccine complications.

	*Cut* *-* *off*	*Sensitivity %*	*Specificity %*	PPV	NPV	*AUC*	*J Value*	*p*
**MELD Score**	>17	71.4 47.8–88.7	70 56.7–83.4	51.7 38.9–64.3	85.4 74.4–92.1	0.753 0.636–0.848	**0.43**	**<0.001**
**Liver Volume**	≤682.3	95.24 76.2–99.9	85.71 72.8–94.1	74.1 58.8–85.1	97.7 86.1–99.7	0.935 0.849–0.980	**0.81**	**<0.001**

Area under the ROC curve (AUC), the 95%CI: (95% confidence interval), Positive predictive value (PPV), negative predictive value (NPV), and J value (Youden’s J value).

**Table 6:** Multivariate logistic regression of potential predictors of COVID-19 vaccine complications.

Covariate	B	SE	Wald	Sig.	RR	95%CI for RR
**ALT (IU/L)**	0.000	0.038	0.000	0.995	1.00	0.93–1.08
**Sodium (mEq/L)**	−0.107	0.107	1.009	0.315	0.90	0.73–1.11
**Creatinine (mg/dL)**	−1.025	2.346	0.191	0.662	0.36	0.00–35.62
**Liver Volume (mL)**	−0.020	0.006	10.485	**0.001**	0.98	0.97–0.99
**MELD**	−0.019	0.198	0.010	0.922	0.98	0.67–1.45
**FIB-4**	0.265	0.269	0.972	0.324	1.30	0.77–2.21
**Constant**	28.035					
Variable(s) entered: ALT, Sodium, Creatinine, Liver Volume, MELD, FIB-4.						

All variables with *p* < 0.1 in a univariate analysis were entered into this regression model. B: regression coefficient; SE: standard error; RR: relative risk; 95%CI: 95% confidence interval, *p* < 0.05 is significant.

## Data Availability

The data presented in this study are available on request from the corresponding author.
